# Examining the synergies and tradeoffs of net-zero climate protection with the Sustainable Development Goals

**DOI:** 10.1177/00368504221138443

**Published:** 2022-12-07

**Authors:** Peter Enevoldsen, Chad M. Baum, Sean Low, Benjamin K. Sovacool

**Affiliations:** 1 Department of Business Development and Technology, Center for Energy Technologies, 1006 Aarhus University, Aarhus, Midtjylland, Denmark; 2 Science Policy Research Unit (SPRU), 1948 University of Sussex Business School, Brighton, UK; 3 Department of Earth and Environment, Boston University, Boston, MA, USA

**Keywords:** Negative emissions technologies, climate change, carbon dioxide removal, solar radiation management, sustainability, energy policy

## Abstract

This article discusses and illuminates the synergies and jeopardies or tradeoffs that exist between the 17 Sustainable Development Goals (SDGs) and net-zero or future climate protection options such as greenhouse gas removal (GGR) technologies and solar radiation management (SRM) deployment approaches, respectively. Through a large-scale expert-interview exercise (N = 125), the study finds firstly that numerous synergies and tradeoffs exist between GGR, SRM, and the SDGs. More specifically, we reveal that GGR deployment could enhance the attainment of 16 of the 17 SDGs, but this comes with possible tradeoffs with 12 of the SDGs. SRM deployment could not only enhance the attainment of 16 of the 17 SDGs, but also create possible tradeoffs with (a different) 12 SDGs. The findings further support the understanding of the complexity of SRM and GGR proposals and help policymakers and industrial pioneers understand, navigate, and benchmark between geoengineering approaches using sustainable development goals.

## Introduction

Limiting global warming to 1.5°C to 2°C above pre-industrial levels is one of the important aspects of the United Nation's 17 Sustainable Development Goals (SDGs). However, climate change has to be viewed as one of the overarching topics requiring intervention, which seeks to improve human lives, ecosystems and inspire and guide policymakers and societal actors. The SDGs can therefore be utilized as a governance framework to benchmark and justify investments and innovations in processes, politics, and technologies. As an example, several studies have pointed toward utilizing mitigation technologies and associated deployment strategies of 100% renewables in 2050 to reduce greenhouse gas emissions, to limit global warming.^1,2^

Despite ongoing political discussions on climate mitigation and innovative behavior in the renewable energy industry,^1^ the existing commitments for climate mitigation are at risk of falling short.^3^ Meanwhile, global greenhouse gas emissions have continued to rise alongside increasingly severe consequences such as irrecoverable carbon in ecosystems^2^ and freshwater evaporation.^4^

It has been documented that early climate action can potentially have multiple benefits, including avoiding hunger, poverty, etc.,^5^ though this also depends on how climate action is undertaken and underlines the requirement of a broad governance framework such as the SDGs. At the same time, given the ongoing trajectory of emissions reductions, increasing attention is being given to how to deal with the prospect of a “climate overshoot.”^3^ In this vein, Anderson and Peters^6^ highlighted that nascent or speculative negative emission technologies are positioned as pivotal for limiting global warming to 2°C, let alone the aim of 1.5°C tentatively agreed upon in the Paris Agreement” - but could represent “an unjust and high stakes gamble”.

Based on temperature observations over 20-year periods merged with emission scenarios it is likely that 2°C will be exceeded during the 21st century,^7^ deeper consideration is being given to other options for intervening in the climate system as well as how these might be undertaken without adversely affecting the welfare of societies, aspirations of human beings, and broader functioning of ecosystems. For this reason, exploring how such climate-intervention technologies intersect with the 17 SDGs is of crucial interest for future research.

The current study employs a large expert-interview exercise (N = 125) to analyze and examine the synergies and tradeoffs of greenhouse gas removal (GGR) technologies and solar radiation management (SRM) deployment for sociotechnical objectives utilizing the 17 SDGs. We rely on a synthesis of recent expert opinions providing an overview of how leading actors in academia and industry perceive these options. Moreover, the recent Intergovernmental Panel on Climate Change report largely focuses on other forms of climate mitigation wherein it discusses the implications for sustainability and connections to the SDGs^5^ and the long-term modeling within scenarios excludes almost all solar geoengineering technologies.^8^ We address this gap by explicitly and systematically connecting multiple forms of carbon removal and solar geoengineering with the SDGs. Lastly, as Denton et al.^5^ also note, modeling often look at climate change mitigation and the SDGs in an aggregate manner, rather than by examining the more granular impacts that may occur at smaller local or sectoral scales. We also address this gap with our original expert data attuned to multiscalar GGR and SRM deployment.

## The need for interdisciplinary sustainability assessments

As described by Minx et al.,^9^ multiple studies have sought to address the negative impacts of climate change by means of the development and deployment of negative emission technologies such as carbon capture and storage (CCS), carbon capture and utilization (CCU), direct air capture (DAC), afforestation, etc., which often are introduced under the terms carbon dioxide removal (CDR) or greenhouse gas removal (GGRs). Furthermore, Minx et al.^9^ found that negative emission technologies are required to decarbonize human activities, thereby complementing existing mitigation strategies, and finally also providing an option for future risk management to reverse climate impacts.

A different suite of solar radiation management (SRM) technologies have been proposed as a potential means to lower global temperatures by reducing the amount of solar irradiance reaching Earth, notably, through stratospheric aerosol injection, marine cloud brightening,^10,11^ or even space-based approaches.^12^ GGR and SRM approaches have been defended as being capable of, and even necessary for, achieving a livable climate, but have also been questioned as distractions from deep-lying decarbonization.^13–15^

Much ongoing assessment of GGR is based on their technical capabilities,^9,16,17^ and the same can be said of SRM.^11,18,19^ Many have called for synthesized social studies and interdisciplinary assessments to help inform policymakers and industries in determining what they might contribute to pathways to limit global warming.^20–23^ Increasing emphasis is also assigned to matters of energy and societal justice.^24–26^

A handful of studies have investigated the potential implications of different kinds of negative emissions technologies for the Sustainable Development Goals.^27–30^ Honegger and colleagues have conducted two foundational studies on the interconnections with and between SDGs – one for SRM,^31^ and the other for GGR.^32^ We build upon these efforts in this paper, which assesses SRM and GGR technologies together in relation to the SDGs. We aim also to add to literatures on the aims and steering effects of the SDGs – these have been found to have more impacts on altering discourse than in engendering policy action and tangible change,^33^ and more assessment is needed on how novel and perhaps controversial climate strategies might demonstrate or erode the worth of this important framework of goals.

## Expert consensus and dissensus to reveal synergies and tradeoffs

An extensive expert-interview exercise was conducted to determine the potential positive SDG synergies and the negative SDG tradeoffs for SRM and GGR, respectively. The outcome of the interviews was mapped and analyzed according to the goals of the SDGs. Previously, the SDGs have been used as a lens to determine long-term global priorities for businesses^34^ and to help orient and set priorities for policymakers and institutions, which have been summarized in Global Goals^35^ with other scientific applications of the SDGs. This framework is also useful as a way to critically assess negative emission technologies, which is underlined by Fuso Nerini et al.^36^ who concluded that “*climate change can undermine 16 SDGs, while combatting climate change can reinforce all 17 SDGs but undermine efforts to achieve 12*.” With regard to our methodology, using semistructured interviews, we ensured that all participants—125 in total—were asked the same set of standard questions, thereby ensuring that their answers on these topics could be compared, while simultaneously leaving the possibility for each conversation to take up interesting new topics and directions that emerged. As such, the interview method proved uniquely suited to gathering insights into the range of viewpoints among the large sample of experts. Statements were selected whenever indicating a significant opinion in regard to the SDG synergies and tradeoffs. Furthermore, interviews offer a robust way to understand and excavate the meaning and importance of particular actions and/or objects, which is therefore valuable in the context of complex topics or events. Concerning climate-intervention technologies and their relationship to the SDGs, the method of expert interviews in specific was deemed important given that, unlike peer-reviewed articles which can take years or months to be published, such interviews afford timely insight into *status quo* understandings (at the time of the interview) of the topic at hand. What is more, as a social and evolving discussion, interviews can evolve into unexpected and potentially fruitful avenues.

The set of experts was selected based on their knowledge about the 20 climate-intervention technology options, determined by whether they had published high-quality, peer-reviewed articles on the topic (2011–2020) or possessed patents and intellectual property related to these technologies. In total, 125 interviews were conducted from May to August 2021, with experts from 104 organizations located in 21 countries, and with 881 years spent working in the geoengineering industry or research community (see Table 1). The respondents were variously engaged at universities and research institutes, civil society and nongovernmental organizations, government and intergovernmental organizations, and/or private sector and industrial associations. The interview consisted of seven broad questions related to the research, development, and potential commercialization of the 10 different negative emissions and 10 different solar geoengineering options. For instance, the interview asked, “What are the synergies and trade-offs of GGR and SRM deployment for other societal objectives and the SDGs?” Given our use of a semistructured interview approach,^37^ this question served as an invitation and jumping-off point for discussions to then proceed in other directions and explore other issues. Annex I provide specific details about all 125 expert participants, while Annex II provides an overview of the questions (and subquestions) asked in the expert-interview exercise. Original data for this study comes largely from Question 3 (on governance) and Question 5 (on sustainability and the SDGs).

**Table 1.** Summary of the demographics of experts who took part in our study.

Summary information	Number
Number of experts	125
Number of organizations represented	104
Number of countries represented	21
Number of academic disciplines represented	34
Cumulative years spent in the geoengineering industry or research community	881
Average years spent in the geoengineering industry or research community	7.8
Number of experts whose current position falls into the following areas:	
Civil society and nongovernmental organizations	12
Government and intergovernmental organizations	8
Private sector and industrial associations	12
Universities and research institutes	94
Number of experts from the Global South*	12

Source: Authors.

Note: The sum equals 126 due to the dual affiliation of one of our experts. *We categorized respondents from the Global South based on the classification provided by Western Pacific Region. Many of these participants were selected from the Solar Radiation Management Governance Initiative (now the Degrees Initiative), which engages with Global South countries and experts.

## Results: synergies and tradeoffs within and between climate protection efforts

This section presents our core results, divided into the four areas of positive synergies between the SDGs and GGR or SRM (respectively), in addition to negative tradeoffs between the SDGs and GGR or SRM.

### Synergizing the SDGs with GGR

As a promising sign, positive synergies between carbon removal and GGR and the SDGs came up in 116 interviews or 92.8% of the time. As explained by one respondent, “*the connections between carbon removal and the SDGs are plentiful, I can envision deployment positively affecting so many, from food security, health of the ocean, human health, and even jobs, these are all the major possible synergies*.” R015 added that “*if we do greenhouse gas removal in a well-governed way, we have only win-win situations with the SDGs*.” R027 spoke about how “*pretty much any GGR technique you can name, you can see that it might be completely benign or result in some real wins for the SDGs*.” R052 concurred that “*if carbon removal is deployed in a thoughtful way, it can work synergistically with a lot of the SDGs*.” R108 commented that “*with CDR, you can create a lot of win-win situations with the SDGs … with agroforestry, you could make food and do CDR together, the system may even be more resilient than if you have a monoculture cropland*.”

**Figure 1.**
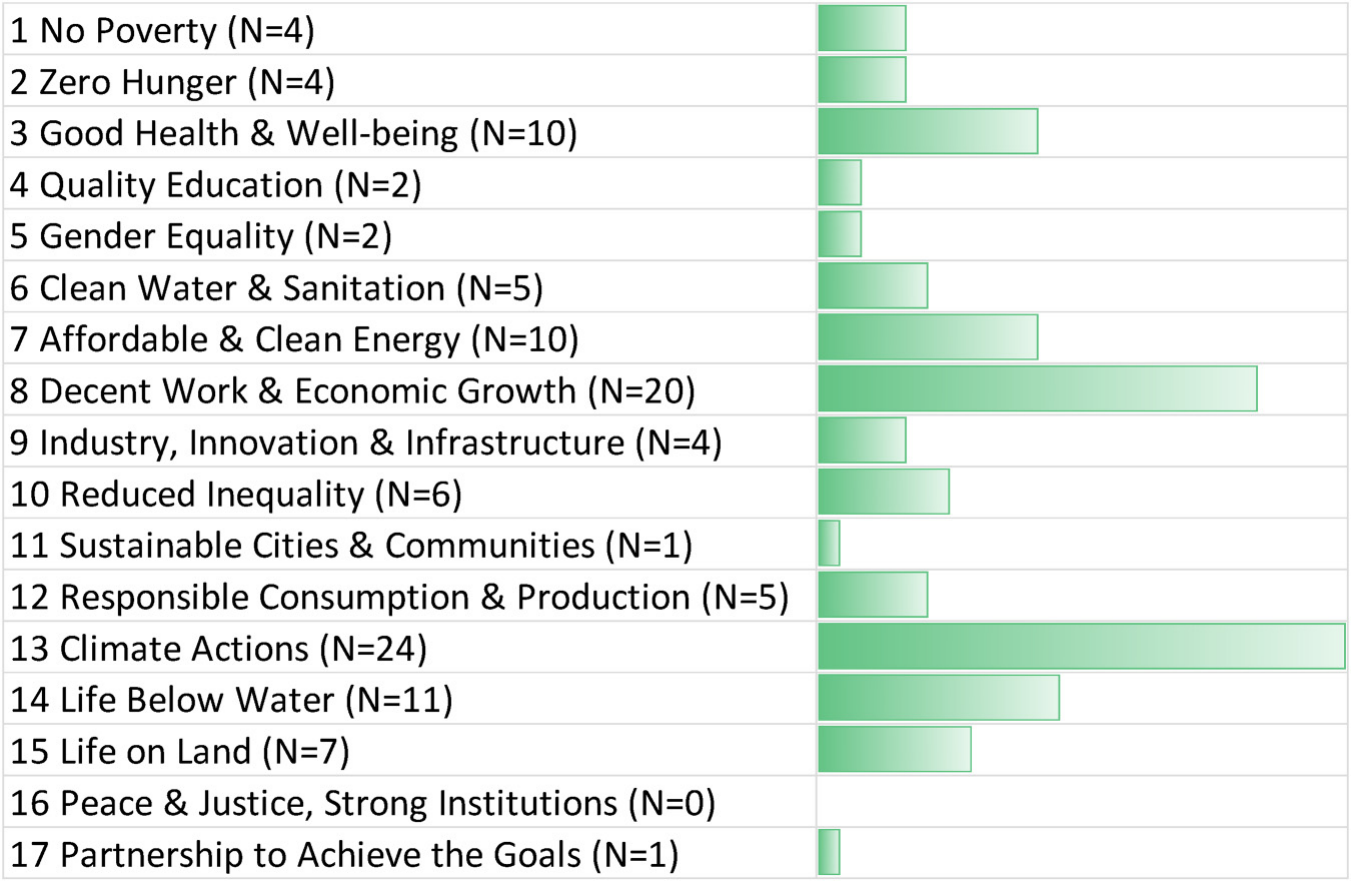
Positive synergies between greenhouse gas removal and the SDGs. Source: Authors, based on expert interviews (N = 125). The bar chart depicts the number of times a positive connection to an SDG was mentioned at least once in an interview. The N for each SDG indicates the number of experts who identified a particular synergy. For more details about our interview sample, see Annex I. SDG: Sustainable Development Goal.

As Figure 1 indicates, the most frequently identified positive connections between GGR and the SDGs related to SDG13 Climate Action (mentioned in 24 interviews, which is unsurprising). However, strong positive linkages were also discussed related to SDG8 (decent work and economic growth), SDG14 (life below water), SDG3 (good health and wellbeing), and SDG7 (affordable and clean energy). Connections to SDG8 centered on the expansion of jobs and employment, especially rural employment, related to large-scale afforestation or bioenergy with carbon capture projects. However, as stated by Intergovernmental Panel on Climate Change,^38^ large-scale afforestation or bioenergy could also negatively impact food security and biodiversity.

Synergies with SDG14 focused on enhanced marine economies and better management of marine protected areas, which could coexist with ocean-based carbon removal. Synergies with SDG3 centered on the improved health outcomes or displaced air pollution or toxic pollution that deployment of negative emissions technologies could attain. Synergies with SDG7 related to the strong coupling of negative emissions systems with bioenergy, solar photovoltaic, solar thermal, wind energy, and even hydrogen and nuclear power technologies. Conversely, few to no positive synergies were identified with SDG16 (peace and justice), SDG17 (partnerships to achieve the goals), and SDG11 (sustainable cities).

The comprehensive and holistic manner in which net-zero can intersect positively with the SDGs was captured by R104. As they stated:

The gap we have currently in energy access and safe water access and access to schooling with gender parity and for ethnic minorities is huge around the world. Distributed, clean energy could be coupled with carbon removal for very large potential synergies for all of those benefits. There's a real, large set of upsides, to work towards universal energy access, universal access to water, food, lighting, IT services, those are things that even without the climate benefit would be huge economic plumes around the world. So, there's a whole set. I mean, it's why the SDGs were designed in the way they were, to have both subcategories to quantify progress, but also a very, very complex map of their linkages across areas. So, the social benefits are really large in making those goals ones that countries are really pushing towards, and climate protection can enhance them all.

R067 concurred and also stipulated that “*for CDR, there are so many co-benefit with the SDGs, including clean energy, land on land, afforestation, and positive impacts on biodiversity, which could be incredibly, really important for sustainability*.”

### Undermining the SDGs with GGR

Unfortunately, negative tradeoffs also were evident within our expert dataset, ones that could seriously jeopardize the attaining of the SDGs if GGR techniques are deployed. Prospective negative tradeoffs were mentioned in 93 interviews or 74.4% of the time. As R052 summarized their perspective, “*the problem with carbon removal is if it's done in a way that's not thoughtful, it could work against all of the SDGs … it's an interesting balance to strike*.” In particular, the interviewee sketched out how negative consequences for land use and food production could cascade across and throughout the various goals.

**Figure 2.**
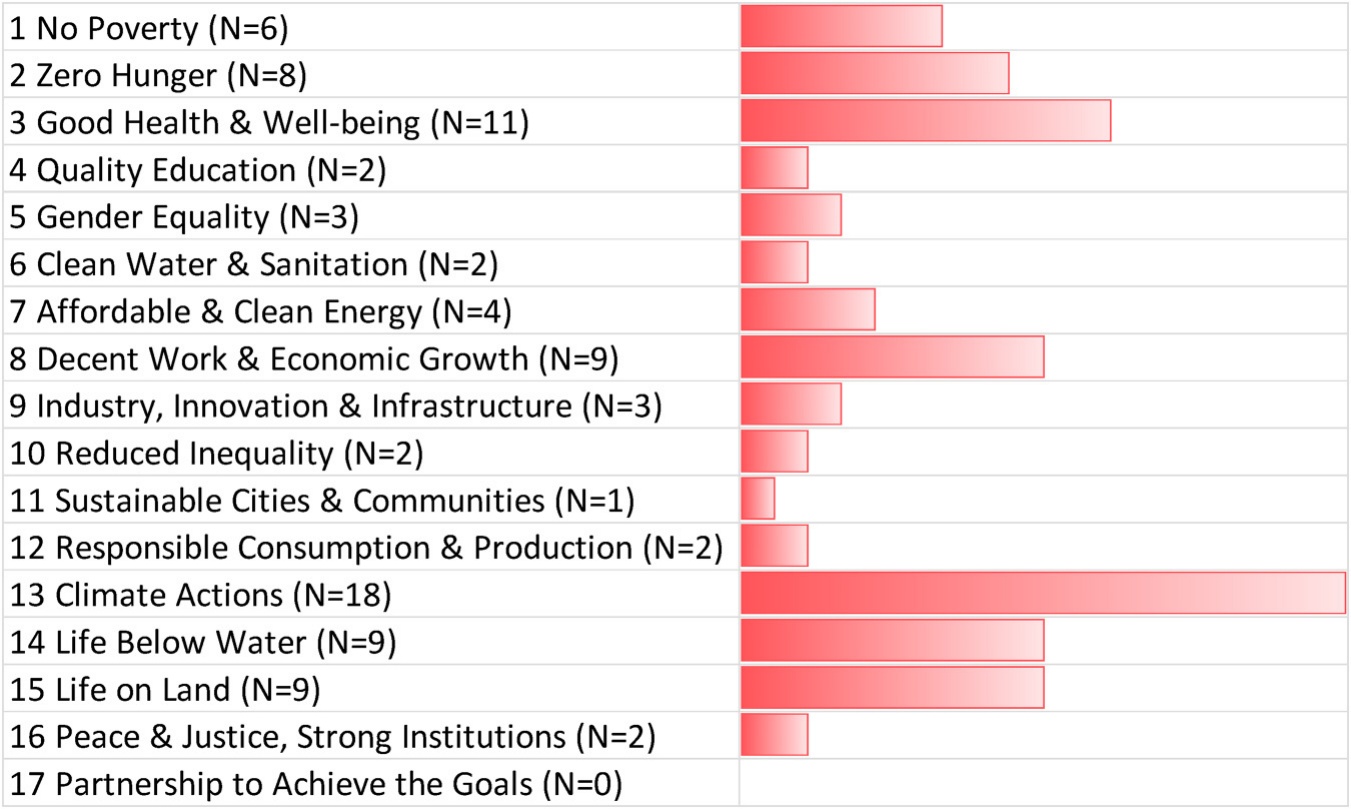
Negative tradeoffs between greenhouse gas removal and the SDGs. Source: Authors, based on expert interviews (N = 125). The bar chart depicts the number of times a negative connection to an SDG was mentioned at least once in an interview. The N for each SDG indicates the number of experts who identified a particular tradeoff. For more details about our interview sample, see Annex I. SDG: Sustainable Development Goal.

As demonstrated by Figure 2, the strongest consensus among experts was that tradeoffs would occur related to SDG2 (zero hunger), SDG14 (life below water), SDG6 (clean water and sanitation), and SDG15 (life on land). Negative connections with SDG2 and SDG6 included disruption to land use, competition over farming, interference with water security and quality, and the dispossession of communities or land-grabbing done via large afforestation or tree plantation projects in the Global South. R093 spoke about how the increased energy needs of carbon removal could have broad environmental impacts across land and water:

If you remove CO2, if it's with DAC or carbon capture and storage, you need energy and this will have a strong negative impact on the environment, on land and water. It cannot be zero. It's impossible. You will maybe improve on climate change, but most likely you will have other impacts, maybe in human health or maybe in ecosystems. Therefore these trade-offs will be there for sure.

R096 added that “*the SDGs on ocean management and marine life are obviously going to be badly impacted if we do massive ocean fertilization or even alkalinization. If we do that on a massive scale, we could devastate ocean ecosystems*.”

### Synergizing the SDGs with SRM

Positive connections with the SDGs were also evident with solar radiation management, which were mentioned in 91 of the interviews (or 72.8% of the time). It should be noted that while several GGR technologies have been deployed in the real world to varying degrees, SRM technologies in general remain rather immature, such that discussions of the respondents on the potential impacts on the SDGs are relatively hypothetical and prospective vis-à-vis their GGR counterparts. R039 articulated that the “*main synergy SRM has with the SDGs is to the extent that they manage to limit climate change … climate change has the potential to devastate all efforts across all 17 SDGs, and that is really the primary purpose in deployment*.” R035 agreed and added that:

If solar geoengineering can reduce global warming and climate change, then the negative effects that we're all aware of—the extreme weather events, the floods, the droughts, the hit on agriculture, the impact on water security, food security, all the rest of it—would be improved. In which case, of course, that would help with all of the Sustainable Development Goals. That's the argument for doing SRM.

R070 similarly argued that “*solar geoengineering would probably have an impact on almost all of these goals set by the United Nations. If you develop these systems in a sustainable way it could impact poverty of course, of course health. If you don’t do anything to the climate all these goals will be impacted very significantly, of course*.”

**Figure 3.**
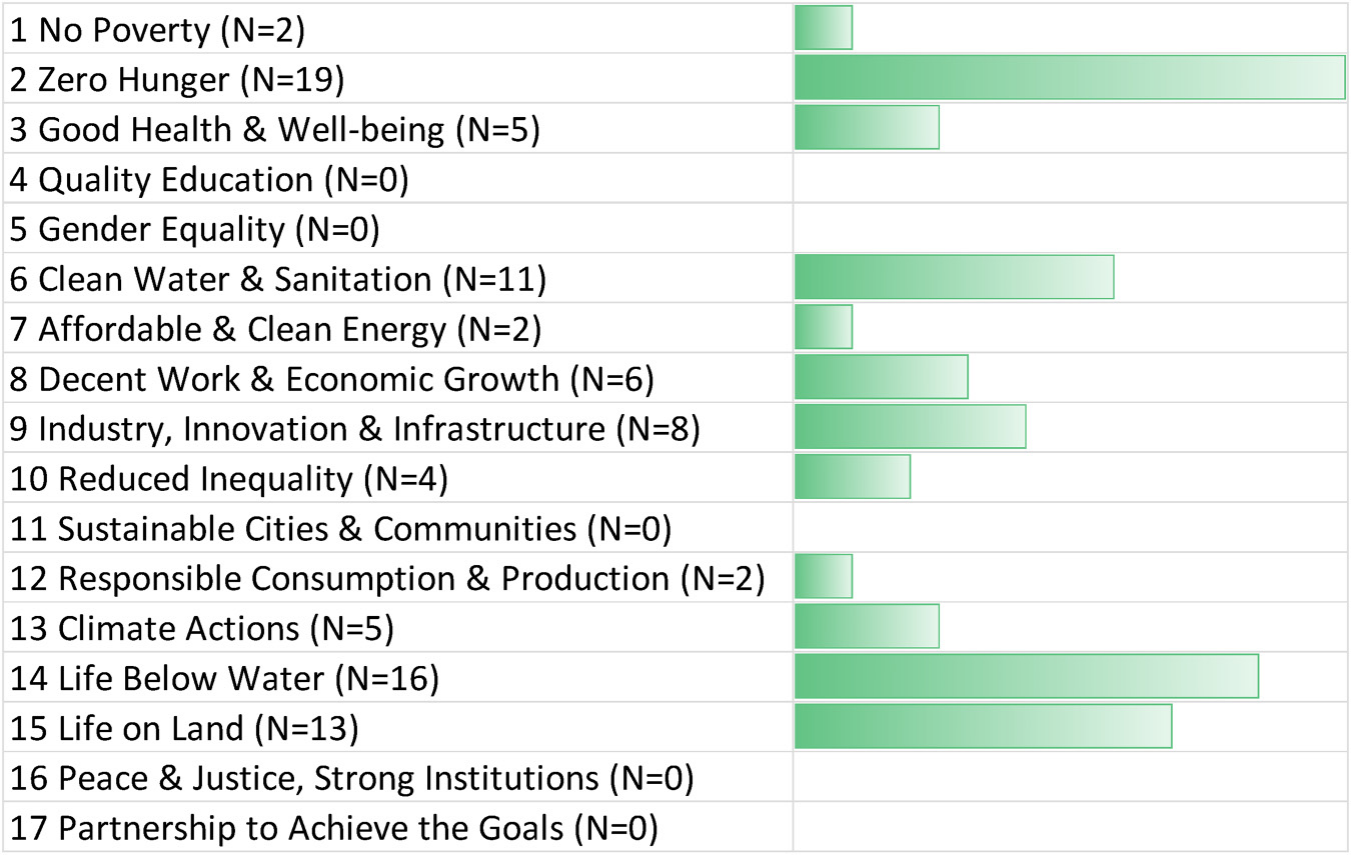
Positive synergies between Solar Radiation Management and the SDGs. Source: Authors, based on expert interviews (N = 125). The bar chart depicts the number of times a positive connection to an SDG was mentioned at least once in an interview. The N for each SDG indicates the number of experts who identified a particular synergy. For more details about our interview sample, see Annex I. SDG: Sustainable Development Goal.

As Figure 3 illustrates, the most mentioned positive synergies related to SDG13 (climate action) but also SDG11 (good health and wellbeing), SDG8 (decent work and economic growth), SDG14 (life below water), and SDG15 (life on land). Good health and wellbeing was seen to connect positively with reduced heat stress. R020 stated that:

Extreme heat will have impacts on health, on agriculture, production, you name it. I mean the biggest challenge right now of the global temperature rise, the direct challenge is already today one of excess heat. There are already many places in the world where because average warming, and then the specific warming in certain areas, which is much higher than the average, cities are becoming unlivable and people are dying or they’re getting sick and that would be reduced or eliminated by SRM. So it has clear impacts on health-related SDGs, on agriculture, food security related SDGs, in a positive sense.

Protection of life below water and above land was connected to reduced temperatures but also more manageable precipitation extremes. R066 explained it as follows:

Precipitation extremes are another major benefit to SRM. Consider Iran and other countries like Afghanistan. Yes, those countries in the region are affected by extreme participation as well, and solar geoengineering is, according to the modelling work, going to reduce those extremes in the region, not only the mean temperatures and mean participation but also extremes, those weather extremes with very high and positive impact on ecosytems and living.

Conversely, few to no connections in Figure 3 were made with SDG17 (partnerships) and SDG11 (cities).

### Undermining the SDGs with SRM

**Figure 4.**
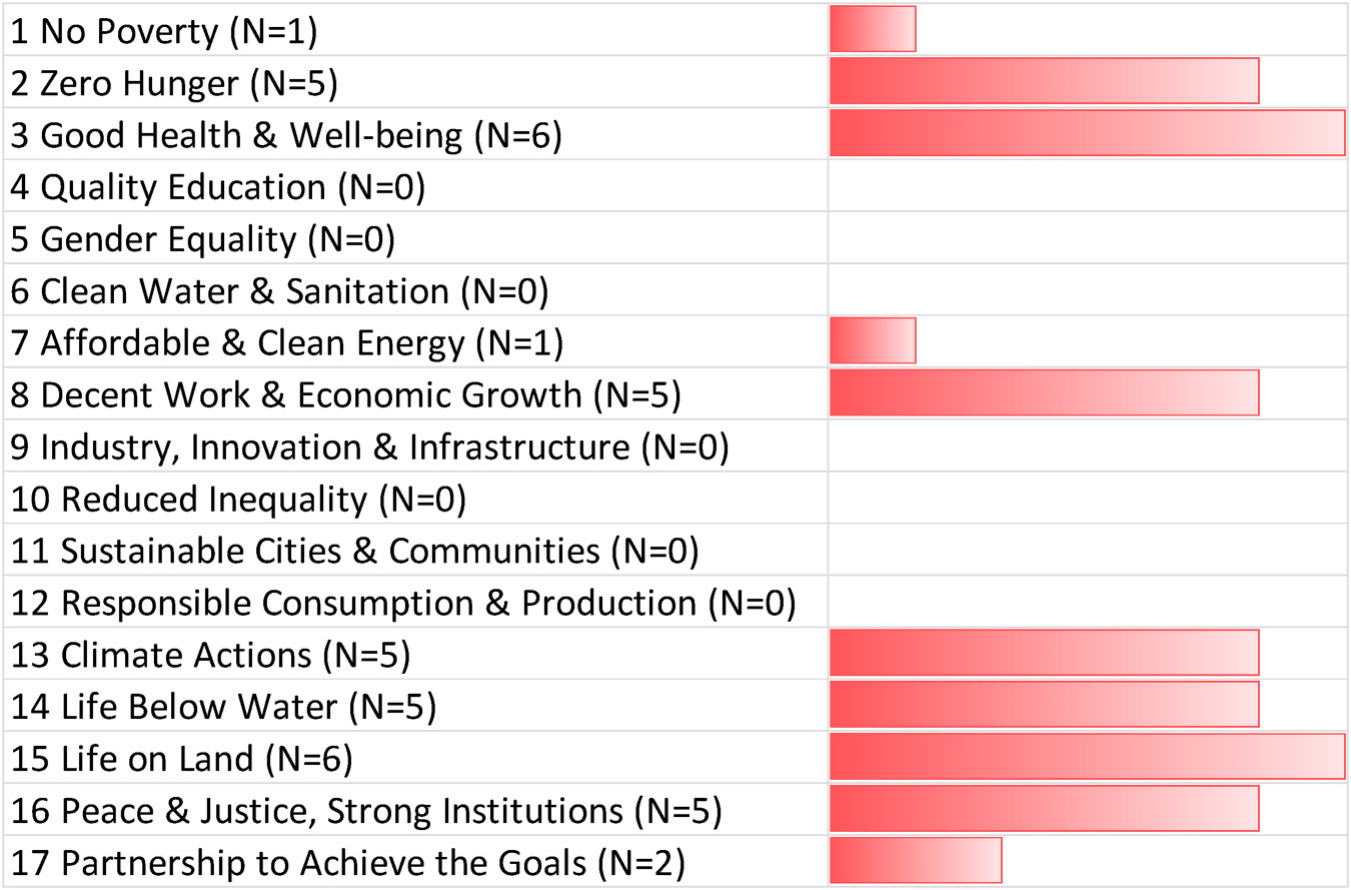
Negative tradeoffs between solar radiation management and the SDGs. Source: Authors, based on expert interviews (N = 125). The bar chart depicts the number of times a negative connection to an SDG was mentioned at least once in an interview. The N for each SDG indicates the number of experts who identified a particular tradeoff. For more details about our interview sample, see Annex I. SDG: Sustainable Development Goal.

Negative connections with SRM and the SDGs arose in 41 interviews or 32.8% of the time. As Figure 4 reveals, the most commonly identified tradeoffs were related to SDG3 (good health and wellbeing) and SDG15 (life on land). R111 captured possible tradeoffs with health and wellbeing by noting that:

If we say SRM is going to reduce rainfall in a particular area, then directly it is going to have negative impacts on agriculture because most of agriculture sub-Saharan African is rainfall dependent. If we change rainfall, we change irrigation and lack of water, and so you have negative impacts on human health, but also community wellbeing, especially among subsistence farmers.

Other expert statements focused on biodiversity and life on land. R036 cautioned that, “*with regard to SRM, the changes to climate could hurt life above land. Dry places would get dryer. Wet places would get wetter. So that would probably accentuate negative ecosystem impacts in some areas*.” R071 stated that solar geoengineering risks “*a termination shock, the risk of suddenly stopping or eventually stopping; fast rates of change of course are really detrimental for ecosystems because they can't move very fast If you have really abrupt changes that you could have by geoengineering, then this could be detrimental to biodiversity*.” R076 similarly expressed concern that “*there is an overlap there with the life on land and SRM, the potential effects of certain choices related to either marine cloud brightening or putting chemicals or introducing plankton growth? That, obviously, will have effects on life above land and below water*.”

## Discussion: nested hierarchies and linkages between the SDGs

**Figure 5.**
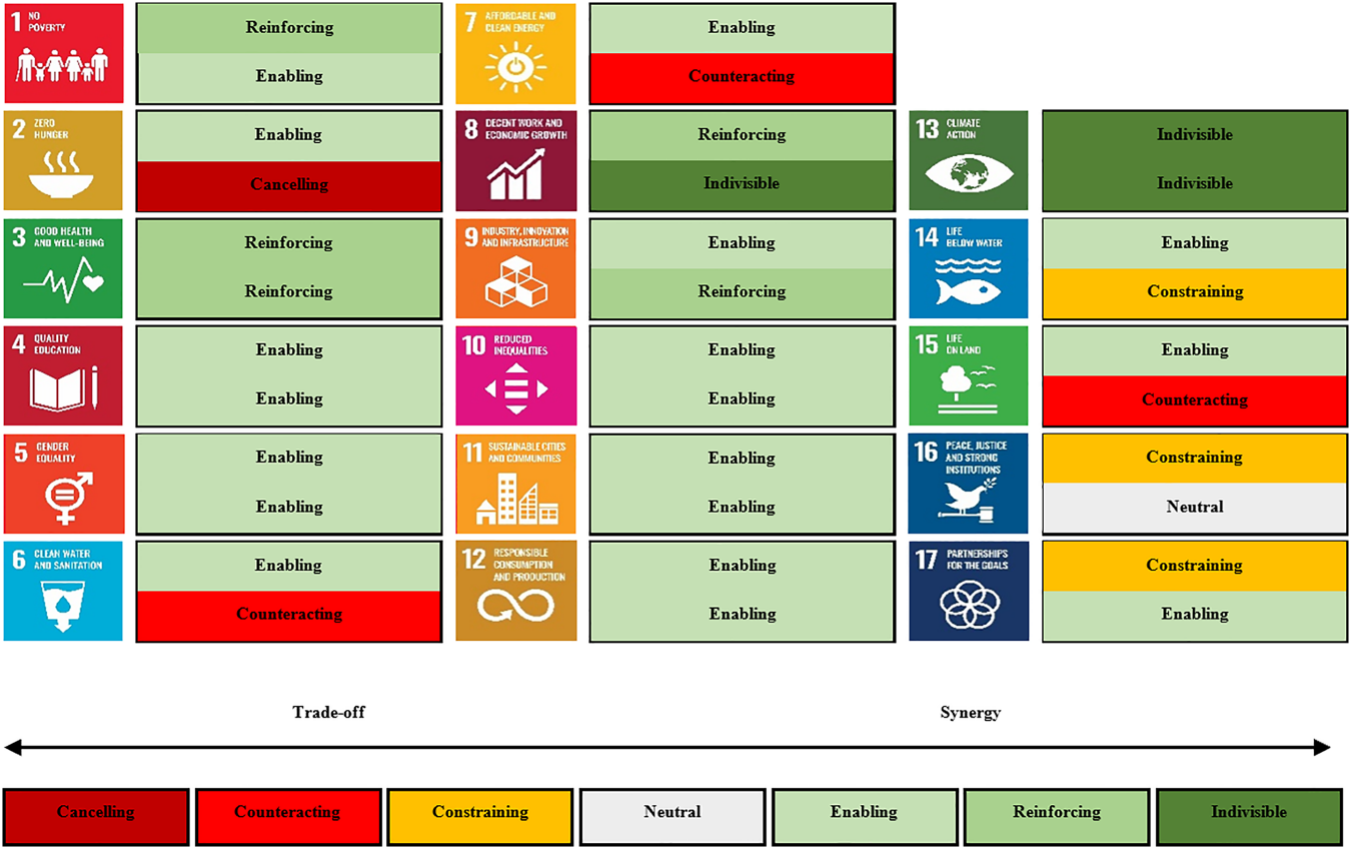
Synergies and tradeoffs with portfolios of GGR and SRM deployment and the Sustainable Development Goals. Source: Authors. The upper box represents SRM while the lower box represents GGR. GGR: greenhouse gas removal; SRM: solar radiation management.

Deployment of GGR and SRM could occur in isolation, but they could also occur together as part of an integrated portfolio. Another way of evaluating impacts is thus not by technology, but by all 17 of the SDGs themselves. In order to establish a more systematic overview of whether SRM and GGR create more tradeoffs or synergies, Figure 5 visualizes our data for both sets of options across all of the SDGs. The division of the impact on each SDG is inspired by the research design from Nilsson et al.,^39^ as it benchmarks SDG synergies and tradeoffs using seven categories to establish a high-level overview of impacts. The division is based on the opinions of the experts where a distribution was conducted based on whether SRM and GGR technologies and solutions would align and enable each SDG, or conflict and cancel. The shortcoming of this approach is in the statistical summarized presentation, as few experts could claim tradeoffs with valid arguments, yet, the output could symbolize a synergy if more experts claimed so. It is therefore sought to highlight such counteracting inputs in the subsequent discussion of Figure 5.

When analyzing the data for the output visualized in Figure 5, it was clear that multiple interactions arise across each of the SDGs with both GGR and SRM. Similarities could be drawn for SRM regarding its relationship with SDG1 to SDG5 and SDG13, as any positive impact on climate action would lead to reduced poverty and disruption of the increasing hunger patterns, which would result in better overall health and living conditions—at least relative to the severe climate-impact scenarios which would be avoided. Fuso Nerini et al.^36^ show that action on climate is a potential multiplier on all others. However, as pointed out by one expert: “*For SRM, we are very far away from knowing how most of the SDGs would be affected*” and even though, e.g., stratospheric aerosol injection was often perceived as having potentially positive impacts for food production in the global south, it was also pointed out that it could adversely influence precipitation patterns or the monsoon, thereby harming local communities and (smallholder) farmers.

The same response pattern was observed for GGR, however, with the main difference being that the experts unanimously claimed that such options would have tradeoffs with hunger (SDG2), clean water (SDG6), and affordable clean energy (SDG7). For food production, the experts pointed out that the land use and water requirements for most GGR technologies, with a specific emphasis on bioenergy with carbon capture and storage (BECCS) and carbon capture and storage technologies in general, would constitute a major tradeoff. At the same time, many experts highlighted the uncertainties and ongoing discussions around how crop yields would be affected by SRM.^40,41^

For DAC, the immediate concern was the extensive energy consumption, among other resources, that would be needed to capture and sequester carbon at the scale required, which was feared to slow down the transition toward a society powered by 100% renewables. Accordingly, one of the recurring points in the discussion was the importance of sitting operations in locations where renewable energy would be sufficiently available, whether this was geothermal energy in Iceland, wind energy in Texas or New Mexico, solar energy in sub-Saharan Africa, and so on—and so that use of fossil-fuel energy would undermine carbon-sequestration potential. In contrast, the endless opportunities of harvesting solar energy were highlighted by several experts with sunshades for SDG7, as sunshades on their own could power all projected energy consumption on Earth by 2050—though, it was noted, this presumes the development of technologies currently far from feasibility.

Experts agreed that GGR could form the core of viable business models for what could be multi-trillion dollar-value sequestration of carbon businesses, with subsequent beneficial impacts for economic growth, industry, and infrastructure (SDG8 and SDG9). In particular, experts pointed to potential opportunities in the marine economy and for the agricultural sector, whereby the ability to grow biomass that could be used for energy or carbon-sequestration purposes could help to stabilize local economies and help to mitigate the negative impacts expected from climate change (SDG1 and SDG2). However, if not properly conceived and monitored, such opportunities could be undercut by risks for injustice and inequality among countries and continents, as the imbalance of distributions of impacts could lead to a global inequality-accelerating system (SDG10 and SDG12). Nevertheless, as pointed out by one of the respondents, “*The consequences of climate changes bring inequality so stopping that will have the opposite effect*.”

Conversely, there were significant doubts about the prospective benefits of SRM for industry and the economy, with only the ability to avoid the worst impacts of climate change tending to be noted. For this reason, the common impression among experts was that such activities would have to be cultivated and incentivized by governments and international multilateral agencies, whether conceived as “public goods” or “waste management.” At the same time, there were notable differences within the set of SRM options, with greater scope envisioned for options like marine could brightening that would have more regionally focused impacts, for instance, to restore coral reefs around Australia and in the Pacific (SDG14), or how the various forms of albedo modification could be used in warmer locales to address growing problems around urban heating (SDG3 and SDG11). Above all, if any of these options would prove useful in avoiding the worst impacts of climate change, as well as those regionally specific challenges highlighted for the Global South (e.g. changing monsoon patterns in southeast Asia, declining precipitation in South America, extreme weather events in the Middle East), then experts were as a whole, and especially those from the Global South, more certain that this would have beneficial impacts not only for climate action (SDG13) but for positively impacting peace and partnership (SDG16 and SDG17), even if only by avoiding the kinds of conflicts and climate-driven mass migrations that can be expected in an ever-warmer world.

For life below water (SDG 14), there was much discussion around the downsides of ocean iron fertilization, given its hypothesized efficacy and role as one of the climate-intervention for dealing with, *inter alia*, the problem of ocean acidification – a role, it must be said, rendered highly fraught by the substantial public blowback and controversy in reaction to prior field trials.^42,43^ In this regard, one expert stressed that “*Climate is a lot more than just temperature*”, illustrating that even if we were to attain the target in the Paris agreement, this would not address all issues, let alone would such an argument serve to guarantee acceptability on the part of the public. Experts were therefore more generally positive about the large-scale reductions possible through ocean alkalinization, if it could be done in a sustainable manner.

Nonetheless, as action in coastal and marine environments tends to evoke higher levels of public concern^20,42^ there was equal skepticism of how the public would respond as well as over the potential, “*to ruin ecosystems just to remove carbon*.” Multiple references to the need for greater attention to and funding for other marine-based options such as blue carbon and growing marine biomass (e.g. kelp and seaweed) to both benefit the climate and provide cobenefits for marine communities. The same arguments were used for life on land (SDG15), as GGR was perceived to suffer from tradeoffs around the prospect of land-grabbing, adverse changes of large-scale (monoculture) plantations on biodiversity and land use, together with questions of where so much carbon would eventually be stored—and how this might impact ecosystems. Conversely, SRM, owing to its purported status as fast, cheap, albeit imperfect.^44^ was perceived to hold synergies in relation to attempts to protect, restore, and promote sustainable use of ecosystems. Interestingly, a respondent highlighted a possible dilemma, and one likely to occupy discussions among policymakers, researchers, and the public in years to come: “…*is your loss of biodiversity greater by having 1% less sunlight or greater by having a 1-degree warmer world?*”

SDG16 and SDG17 were considered general prerequisites for implementing large-scale SRM and GGR solutions, respectively. However, the experts had concerns over the risks associated with weaponization and misuse of SRM approaches in specific, for instance, as articulated by a respondent, about the need to “*create global well-functioning institutions to actually govern geoengineering*.”

## Conclusion: governing complex interactions in future deployment

Admittedly, not all of our experts were as definitive or supportive of connecting GGR or SRM deployment with the SDGs. R047 spoke about how the SDGs are less a concrete set of targets and more a broad reflection of what society wants to do

To me, the SDGs are totally orthogonal to geoengineering or carbon removal. I think the SDGs are a reflection of what society says it wants to do. That the MDGs were so successful that everyone got into the business of target setting. Then we produced the SDGs, which was just a proliferation of goals. Some of them are real; some of them aren't real. It's therapeutic. It's better than alcoholism, I suppose. Some of them are now backed by real programs, trying to figure out and measure progress. But others are just vague ambitious, and I am not sure they offer a useful platform for action.

Moreover, not all of our experts were sanguine or certain about the direction of impacts between net-zero and the SDGs. R063 spoke about the deep uncertainty in even knowing what effect deployment will have on the SDGs, as well as how whether they synergize or jeopardize is conditional on governance:

The deep systemic nature of the sustainable development goals and the artificial construct around the metrics you need to hit them and then the deep uncertainty regarding CDR and it's development and the way they interact. It means that any quantification or assessment is largely arbitrary … They almost certainly do interact. But the extent of which will be realized as and if we develop the CDR sector going forward is highly uncertain.

R071 elaborated on this theme as well, noting that far more complex analysis is needed before the community can say with certainty a negative or positive impact will occur:

The risks with GGR and SRM of course also impact SDGs. It’s actually a rather complex analysis. If you just do mitigation, how do you impact SDGs? It’s really hard. If you do geoengineering, it’s even harder because you suddenly have several levers and you have several uncertainties. Right now, I think we’re just starting to poke at this and we don’t really have the tools to do it, which gives us another point on research needs: how health impacts are being impacted by mitigation, adaptation, geoengineering and carbon capture is somewhat unclear. We have some ideas, but we’re mostly clueless.

That said, other experts did discuss how under the right governance conditions SDG action can be synergized and tradeoffs minimized and avoided. R065 explained it as follows:

Some of the GGR or SRM technological options can have benefits in addition to climate, if they increase your highly skilled workforce and provide jobs, like that, for instance. Again, it's hard to boil down to single sentence answers. I think it's technology and context specific. It's always possible to do things really, incredibly badly and screw them up. So, it's very easy to talk about the risks depending on your kind of outlook on things. I think there's scope for positives as well as negatives on almost all fronts, in the right context. Context is king.

R099 agreed and argued that whether the SDGs are attained or eroded “*depends on if they work, if they work then the technologies are fixing the climate problem, and you have huge positive feedbacks, of course, with many of the SDGs. And if you mess it up in the one or the other way, then you have huge negative impacts, like land, food, and water*.”

These expert statements underscore that both GGR and SRM require complex adaptive management to preempt risks and tradeoffs and capture synergies and benefits.^45–47^ This is inherently evident in our earlier analysis as well. Our Results reveal a consensus from our expert data that GGR use has the greatest potential to synergize SDG7 (climate action) and SDG8 (decent work and economy work). But our Discussion and Conclusion shows how a similar consensus among experts exists that GGR use will have negative tradeoffs with SDG2 (zero hunger) and SDG14 (life below water). Two sets of SDGs are pitted against each other. Similarly, some of our Results reveal broad beliefs among experts that SRM deployment can synergize with SDG 7 (climate action) and SDG 3 (good health and well-being), some of our other Results indicate diverse reasons for concern, specifically that it could trade-off with SDG 15 (life on land) and also SDG 3 (good health and well-being). Here we even see a direct internal trade-off within one specific SDG concerning jobs and employment versus poverty and dispossession. In simpler terms: it may very well be that GGR technologies are able to provide benefits for decent work and economic growth at the expense of eroding food security and aggravating hunger, while SRM could potentially help to alleviate threats from climate change while elsewhere exacerbating risks to life on land and elsewhere.

Indeed, the need for forums where the kinds of necessary discussions around whether, and where, to deploy particular technologies—or what kinds of portfolios may be most viable—is notable for its absence right now, as also demonstrated by recent disagreement and controversy around a nonuse agreement on geoengineering.^48,49^ This also points to a key research gap. Our interview data did not reveal any consensus around the key factors behind the positive and negative linkages to the SDGs. Future research should explore the key mechanisms and causal linkages behind the synergies and tradeoffs identified by our study.

Nevertheless, this gap only makes the need to systematically guide such debates and resulting causal impacts on deployment, more meaningful and urgent. Because climate change itself impinges on every single SDG, interventions attempting to address it touch on them as well. Future researchers and planners need to understand the full menu of risks and opportunities inherent in GGR and SRM—recognizing prospective synergies, but also realizing the danger of recurring tradeoffs and jeopardies. Whether future climate protection supports or undermines the attainment of the SDGs—whether offers a vehicle of focused improvement, or an engine of detrimental postponement—remains to be determined.

## Annex I: List of 125 semistructured expert interview respondents.

**Table:**

Name	Actor type	Gender	Country	Institution
[Anonymous Aerospace Engineer]	Private Sector + Industrial Associations	Male	Germany	[Aerospace and space systems company focusing on integrated spacecraft]
Aganaba, Timiebi	Universities + Research Institutes	Female	The United States	Arizona State University
Asayama, Shinichiro	Government + Intergovernmental Organizations	Male	Japan	National Institute for Environmental Studies
Bauer, Christopher Dean 'Casey'	Private Sector + Industrial Associations	Male	The United States	Raytheon Space and Defense
Bazilian, Morgan	Universities + Research Institutes	Male	The United States	Colorado School of Mines
Bellamy, Rob	Universities + Research Institutes	Male	The United Kingdom	University of Manchester
Beuttler, Christoph	Private Sector + Industrial Associations	Male	Switzerland	Climeworks
Biermann, Frank	Universities + Research Institutes	Male	The Netherlands	Utrecht University
Boettcher, Miranda	Universities + Research Institutes	Female	Germany	Institute for Advanced Sustainability Studies (IASS)
Brauer, Uwe	Private Sector + Industrial Associations	Male	Germany	Planetary Sunshade Foundation
Brickett, Lynn	Government + Intergovernmental Organizations	Female	The United States	Department of Energy
Briggs, Chad	Universities + Research Institutes	Male	The United States	University of Alaska, Anchorage
Brown, Marilyn	Universities + Research Institutes	Female	The United States	Georgia Institute of Technology
Bruce, John	Private Sector + Industrial Associations	Male	Canada	Carbon Engineering
Buck, Holly Jean	Universities + Research Institutes	Female	The United States	University at Buffalo
Burns, Wil	Universities + Research Institutes	Male	The United States	American University
Caldeira, Ken	Universities + Research Institutes	Male	The United States	Breakthrough Energy, Carnegie Institution for Sciences, and Stanford University, and Stanford University
Camilloni, Ines	Universities + Research Institutes	Female	Argentina	University of Buenos Aires (and Harvard University)
Carton, Wim	Universities + Research Institutes	Male	Sweden	Lund University
Centers, Ross	Private Sector + Industrial Associations	Male	Germany	Planetary Sunshades
Chalecki, Beth	Universities + Research Institutes	Female	The United States	University of Nebraska Omaha
Chavez, Anthony E.	Universities + Research Institutes	Male	The United States	Northern Kentucky University
Clarke, Leon	Universities + Research Institutes	Male	The United States	University of Maryland
Clarke, William S. (Sev)	Private Sector + Industrial Associations	Male	Australia	Winwick Business Solutions
Cobo Gutiérrez, Selene	Universities + Research Institutes	Female	Switzerland	ETH Zurich
Cox, Emily	Universities + Research Institutes	Female	The United Kingdom	Cardiff University
Creutzig, Felix	Universities + Research Institutes	Male	Germany	Mercator Research Institute on Global Commons and Climate Change (MCC)
Delina, Laurence	Universities + Research Institutes	Male	Hong Kong	Hong Kong University of Science and Technology
Di Marco, Leon	Private Sector + Industrial Associations	Male	The United Kingdom	FSK Technology Research—Consultant
Dooley, Kate	Universities + Research Institutes	Female	Australia	University of Melbourne
Draper, Kathleen	Civil Society	Female	The United States	International Biochar Initiative
Elliott, David	Universities + Research Institutes	Male	The United Kingdom	The Open University
Erbay, Yorukcan	Private Sector + Industrial Associations	Male	The United Kingdom	Element Energy
Felgenhauer, Tyler	Universities + Research Institutes	Male	The United States	Duke University
Florin, Marie-Valentine	Universities + Research Institutes	Female	Switzerland	EPFL International Risk Governance Center (IRGC)
Forster, Piers	Universities + Research Institutes	Male	The United Kingdom	University of Leeds
Frumhoff, Peter	Civil Society	Male	The United States	Union of Concerned Scientists
Fuhrman, Jay	Government + Intergovernmental Organizations	Male	The United States	Pacific Northwest National Laboratory (PNNL)
Fuss, Sabine	Universities + Research Institutes	Female	Germany	Mercator Research Institute on Global Commons and Climate Change (MCC)
Gambhir, Ajay	Universities + Research Institutes	Male	The United Kingdom	Imperial College London
Geden, Oliver	Government + Intergovernmental Organizations	Male	Germany	German Institute for International and Security Affairs (SWP)
Ghosh, Arunabha	Civil Society	Male	India	Council on Energy, Environment and Water (CEEW)
Grant, Neil	Universities + Research Institutes	Male	The United Kingdom	Imperial College London
Gruebler, Arnulf	Universities + Research Institutes	Male	Austria	International Institute for Applied Systems Analysis (IIASA)
Guillen Gosalbez, Gonzalo	Universities + Research Institutes	Male	Switzerland	ETH Zurich
Haberl, Helmut	Universities + Research Institutes	Male	Germany	BOKU Vienna
Haigh, Joanna	Universities + Research Institutes	Female	The United Kingdom	Imperial College London / Grantham Institute
Hamilton, Clive	Universities + Research Institutes	Male	Australia	Charles Stewart University
Hartmann, Jens	Universities + Research Institutes	Male	Germany	University of Hamburg
Hawkes, Adam D.	Universities + Research Institutes	Male	The United Kingdom	Imperial College London
Healey, Peter	Universities + Research Institutes	Male	The United Kingdom	Oxford University
Heap, Richard	Civil Society	Male	The United Kingdom	Carbon Removal Centre, Foresight Transitions
Hepburn, Cameron	Universities + Research Institutes	Male	The United Kingdom	Oxford University
Herzog, Howard	Universities + Research Institutes	Male	The United States	MIT
Heyen, Daniel	Universities + Research Institutes	Male	Germany	TU Kaiserslautern (formerly ETHZ)
Heyward, Clare	Universities + Research Institutes	Female	Norway	UiT—the Arctic University of Tromso
Honegger, Matthias	Universities + Research Institutes	Male	Germany	Institute for Advanced Sustainability Studies (IASS)
Horton, Joshua B.	Universities + Research Institutes	Male	The United States	Harvard University
Irvine, Pete	Universities + Research Institutes	Male	The United Kingdom	UCL
Jinnah, Sikina	Universities + Research Institutes	Female	The United States	UC Santa Cruz
Johnson, Les	Government + Intergovernmental Organizations	Male	The United States	NASA Marshall Space Flight Center
Kammen, Daniel	Universities + Research Institutes	Male	The United States	UC Berkeley
Karami, Khalil	Universities + Research Institutes	Male	Slovenia/Germany	University of Ljubljana/University of Leipzig
Karlsberg Schaffer, Madeleine	Civil Society	Female	The United States	SilverLining
Keller, David	Universities + Research Institutes	Male	Germany	GEOMAR—Helmholtz Centre for Ocean Research Kiel
Keller, Klaus	Universities + Research Institutes	Male	The United States	Penn State University
Kravitz, Ben	Universities + Research Institutes	Male	The United States	Indiana University
Kruger, Tim	Private Sector + Industrial Associations	Male	The United Kingdom	Origen Power
Kuswanto, Heri	Universities + Research Institutes	Male	Indonesia	Institut Teknologi Sepuluh Nopember
Lawrence, Mark	Universities + Research Institutes	Male	Germany	Institute for Advanced Sustainability Studies (IASS)
Lehmann, Johannes	Universities + Research Institutes	Male	The United States	Cornell University
Lenton, Andrew	Government + Intergovernmental Organizations	Male	Australia	CSIRO
Lin, Albert	Universities + Research Institutes	Male	The United States	UC Davis
MacMartin, Doug	Universities + Research Institutes	Male	The United States	Cornell University
Mahajan, Aseem	Universities + Research Institutes	Male	The United States	Harvard University
Malik, Abdul	Universities + Research Institutes	Male	Saudi Arabia	King Abdullah University of Science and Technology (formerly Grantham Institute)
McLaren, Duncan	Universities + Research Institutes	Male	The United Kingdom	Lancaster University
Mengis, Nadine	Universities + Research Institutes	Female	Germany	GEOMAR—Helmholtz Centre for Ocean Research Kiel
Merk, Christine	Universities + Research Institutes	Female	Germany	Kiel Institute for the World Economy
Michaelowa, Axel	Universities + Research Institutes / Private Sector + Industrial Associations	Male	Switzerland	University of Zurich / Perspectives Climate Group
Montserrat, Francesc	Universities + Research Institutes	Male	The Netherlands	Project Vesta / Royal Boskalis Westminster N.V.
Moore, John	Universities + Research Institutes	Male	Finland	University of Lapland / Arctic Centre
Moreno-Cruz, Juan	Universities + Research Institutes	Male	Canada	University of Waterloo
Morrow, David	Universities + Research Institutes	Male	The United States	American University
Muri, Helene	Universities + Research Institutes	Female	Norway	Norwegian University of Science and Technology (NTNU)
Obersteiner, Michael	Universities + Research Institutes	Male	The United Kingdom	Oxford University
Odoulami, Romaric	Universities + Research Institutes	Male	South Africa	University of Cape Town
Parker, Andy	Civil Society	Male	The United Kingdom	SRM Governance initiative
Parson, Edward 'Ted' A.	Universities + Research Institutes	Male	The United States	UCLA
Pasztor, Janos	Civil Society	Male	Switzerland	Carnegie Climate Governance Initiative
Pidgeon, Nick	Universities + Research Institutes	Male	The United Kingdom	Cardiff University
Pinto, Izidine	Universities + Research Institutes	Male	South Africa	University of Cape Town
Pongratz, Julia	Universities + Research Institutes	Female	Germany	University of Munich
Preston Aragonès, Mark	Civil Society	Male	Norway	Bellona Foundation
Rahman, Mohammed Mofizur	Universities + Research Institutes	Male	Germany	TH Cologne—University of Applied Sciences
Raimi, Kaitlin T.	Universities + Research Institutes	Female	The United States	University Michigan
Reiner, David	Universities + Research Institutes	Male	The United Kingdom	Cambridge University
Renforth, Phil	Universities + Research Institutes	Male	The United Kingdom	Heriot-Watt University
Reynolds, Jesse	Universities + Research Institutes	Male	The United States /The Netherlands	UCLA/Independent Consultant
Rickels, Wilfried	Universities + Research Institutes	Male	Germany	Kiel Institute
Robock, Alan	Universities + Research Institutes	Male	The United States	Rutgers University
Rothman, Dale	Universities + Research Institutes	Male	The United States	University of Denver
Rouse, Paul	Universities + Research Institutes	Male	The United Kingdom	University of Southampton
Schleussner, Carl	Civil Society	Male	The United States	Climate Analytics
Schmidt, Joern	Universities + Research Institutes	Male	Germany	Kiel Institute
Schneider, Linda	Civil Society	Female	Germany	Heinrich Boell Foundation
Scott, Vivian	Universities + Research Institutes	Male	The United Kingdom	Edinburgh University
Simonelli, Lucia	Civil Society	Female	The United States	Carbon 180
Smith, Pete	Universities + Research Institutes	Male	The United Kingdom	University of Aberdeen
Smith, Steve	Universities + Research Institutes	Male	The United Kingdom	Oxford University
Smith, Wake	Universities + Research Institutes	Male	The United States	Harvard University
Spangenberg, Joachim	Universities + Research Institutes	Male	Germany	Sustainable Europe Research Institute SERI Germany e.V
Stephens, Jennie	Universities + Research Institutes	Female	The United States	Northeastern University
Stoefs, Wijnand	Civil Society	Male	Belgium	Carbon Market Watch
Sugiyama, Masahiro	Universities + Research Institutes	Male	Japan	University Tokyo
Sunny, Nixon	Universities + Research Institutes	Male	The United Kingdom	Imperial College London
Surprise, Kevin	Universities + Research Institutes	Male	The United States	Mount Holyoke College
van Vuuren, Detlef	Government + Intergovernmental Organizations	Male	The Netherlands	PBL Netherlands Environmental Assessment Agency
Vaughan, Nem	Universities + Research Institutes	Female	The United Kingdom	University of East Anglia
Victor, David	Universities + Research Institutes	Male	The United States	UC San Diego
Vivian, Chris	Government + Intergovernmental Organizations	Male	The United Kingdom	GESAMP
Wagner, Gernot	Universities + Research Institutes	Male	The United States	NYU
Wolske, Kimberly S.	Universities + Research Institutes	Female	The United States	University Chicago
Wood, Robert	Universities + Research Institutes	Male	The United States	University of Washington
Workman, Mark	Universities + Research Institutes	Male	The United Kingdom	Energy Futures Lab, Imperial College London

## Annex 2: Full GENIE semistructured expert interview question and subquestion sets

**Table:**

**Topic**	**Central question**	**Subquestions**
**1. Innovation**	Which particular options have high or low innovation potential in technical, communication, societal appraisal, and policy dimensions?	What are critical innovation gaps? Which particular options have high or low learning potential? What is the potential for massive scale up or scaling?
**2. Coupling**	What energy systems or other sociotechnical systems could or should be coupled to carbon removal?	What are complementary (or alternate) technologies and/or upstream and downstream technologies (or systems) a la the Technology Environment Analysis? In what ways might greenhouse gas removal or solar radiation management tradeoff with, or further strengthen, renewable energy deployment?
**3. Business models**	What business models and markets could carbon removal create or disrupt?	Do you see the technologies benefitting new entrants, or incumbents? Who is financing development or deployment? What policies or changes in governance are needed?
**4. Risks**	Which serious risks (e.g. social, political, military, ethical, and environmental) may arise?	What are possible risks for climate mitigation or adaptation? What about the risk of accidents? Is there a risk of weaponization?
**5. Sustainability**	What are the synergies and tradeoffs of deployment for the Sustainable Development Goals and other societal objectives?	On balance are there more synergies or tradeoffs? Which are the most important?
**6. Justice**	What vulnerable groups could be affected, positively or negatively?	Who is most vulnerable? What about impacts on indigenous groups? What about future generations?
**7. Actors**	Who are the relevant (or most important) actors (or stakeholders / networks), for example, commercialization, development, and/or acceptability?	Who are the most powerful actors? Are there any missing or invisible actors? What about the military, or other nontraditional or subnational actor networks?

Source: Authors.
